# Effect of the Interaction Between Dietary Fiber Structure and Apparent Viscosity on the Production Performance of Growing Pigs

**DOI:** 10.3390/ani15223310

**Published:** 2025-11-17

**Authors:** Feng Yong, Huijuan Li, Bing Hu, Bo Liu, Rui Han, Dongsheng Che

**Affiliations:** 1 Key Laboratory of Animal Production, Product Quality and Security, Ministry of Education, Changchun 130118, China; yongfeng0485@163.com (F.Y.); js9794lyy@163.com (H.L.); 18243829340@163.com (B.H.); 13166884099@163.com (B.L.); 2 Jilin Provincial Key Laboratory of Animal Nutrition and Feed Science, Changchun 130118, China; 3 Jilin Provincial Science and Technology Innovation Center of Pig Industry Technology, Changchun 130118, China; 4 College of Animal Science and Technology, Jilin Agricultural University, Changchun 130118, China

**Keywords:** dietary fiber structure, apparent viscosity, growth performance, carcass traits, meat quality, intestinal microbiota, pigs

## Abstract

To investigate the regulatory effects of dietary fiber structure (β-glucan-to-arabinoxylan ratio, β/AX) and apparent viscosity (AV) on production performance in pigs, this study used a 2 × 3 factorial design, randomly assigning 36 growing pigs (47.2 ± 1.5 kg) to six dietary treatments (two AV levels and three β/AX ratios), and observed the growth performance, carcass traits, meat quality, intestinal microbiota, and liver lipid metabolism. The results showed that increased dietary β/AX and AV reduced subcutaneous fat deposition, improved meat tenderness and the nutrient content of meat, but decreased pig weight gain and dressing percentage. Increased dietary β/AX and AV selectively promoted the relative abundance of butyrate-producing bacteria and the concentration of butyrate in the middle colon, thereby regulating the expression of genes related to hepatic de novo lipid synthesis and oxidation, reducing serum glucose and total cholesterol levels, and increasing plasma glucagon-like peptide-1. These findings reveal the potential mechanism by which the physicochemical properties of dietary fiber mediate lipid metabolism to reduce weight gain and provide new insights for regulating fat deposition in pigs by controlling the structural and physical properties of dietary fiber.

## Simple Summary

Increasing the feeding proportion of unconventional feeds used to replace grain-based feeds in conventional diets has become an important goal for improving the economic efficiency and sustainable production strategies of the pig industry. However, the number of unconventional feeds used is limited due to their high dietary fiber content. How to improve the utilization efficiency of high-fiber feeds and diets in pig production is an urgent issue that needs to be addressed nowadays. This study found that under conditions of relatively high dietary fiber levels, differences in fiber structure and apparent viscosity significantly affect the growth performance, carcass traits, meat quality, and gut microbiota of growing pigs. These findings provide a theoretical basis for the effective utilization and rational combination of agricultural by-products.

## 1. Introduction

The pig industry and the formation of its products play an important role in the development of the global economy and the guarantee of food security. With the improvement of the overall scale and productivity of the pig industry, there is growing interest in how to use low-cost unconventional feeds to formulate diets that meet the maintenance and production needs of pigs, so as to increase the output per unit input [1,2]. However, due to their high dietary fiber (DF) content, the proportion of these feeds in the complete diets of pigs can only be used at a limited ratio [3,4]. Studies have shown that appropriately increasing dietary fiber levels can improve pigs’ feed conversion rate and growth performance [5], while also regulating the enhancement of animal welfare and intestinal health [6,7]. For example, a moderate increase in dietary fiber exerts positive effects on enhancing pigs’ satiety, reducing stereotypic behaviors, promoting chyme emptying in the intestinal lumen, and alleviating intestinal inflammatory responses [8,9,10]. However, when the proportion of fibrous agricultural and sideline by-products in the diet is further increased, that is, when dietary fiber levels are raised further, it often reduces the effective nutrient concentration of the diet and pigs’ production performance [11,12]. Therefore, how to improve the utilization rate of unconventional feeds in pig production without additional feed costs is not only an old issue, but also an important problem that needs to be solved urgently at present.

As the main component of the cell wall of plant crops and their by-products, DF is a class of carbohydrate polymers with multiple properties (nutritive, non-nutritive, and anti-nutritive), and its nutritive property is mainly manifested under the action of intestinal microorganisms. It is currently clear that except for a few DFs (some oligosaccharides and resistant starches) which are degraded at the end of the small intestine, most of them are fermented under the action of hindgut microorganisms to produce metabolites such as short-chain fatty acids, thereby providing energy for the animal [13,14]. The anti-nutritive property of DF usually appears when its level is too high, which is reflected in reducing the effective nutrients of feed and the production performance of animals [11]. The non-nutritive effect of DF is mainly determined by its physical properties, such as solubility and apparent viscosity [15,16]. Recent studies have shown that the fermentability of DF is related to its solubility. For example, in vitro fermentation experiments using fiber mixtures with different ratios of soluble dietary fiber (SDF) to insoluble dietary fiber (IDF), composed of purified inulin and non-starch polysaccharide mixtures, found that the fermentability of fiber increases with the increase in the proportion of the SDF [17]. However, this finding is not completely consistent with the results obtained from animal experiments. Studies have found that the concentration of short-chain fatty acids in the feces of growing pigs is positively correlated with the digestibility of IDF, but has no correlation with the digestibility of SDF, suggesting differences between purified DF and natural feed-derived DF [18]. Compared with many studies on purified DF, our previous studies have confirmed that the chemical structure (β-glucan-to-arabinoxylan ratio, β/AX) and apparent viscosity (AV) of natural feed-derived DF play key roles in regulating its fermentation efficiency and affecting the energy metabolism process of animals [19]. However, the effects of differences in DF structure and apparent viscosity on the growth performance and product quality of pigs, as well as their mechanisms, remain unclear.

Therefore, this study aims to investigate the effects of differences in DF structure and apparent viscosity on the growth performance, carcass traits, meat quality, and intestinal microbiota of growing pigs under the condition of a relatively high DF supply level. It is expected to provide a reference for the application of unconventional feeds in pig production and the feeding strategy of high DF diets.

## 2. Materials and Methods

### 2.1. Ethics Statement

Under the Guidelines for the Care and Use of Experimental Animals from Jilin Agricultural University, we carried out all animal tests. These activities received approval from the Ethics Committee of Jilin Agricultural University (approval number: KT2024001).

### 2.2. Experimental Design and Animals

We employed a 2 × 3 two-factor factorial treatment arrangement. The primary factors under investigation were two dietary levels of AV and three ratios of dietary β-glucan-to-arabinoxylan (β/AX). We set 36 growing pigs (Duroc × Landrace × Yorkshire) with comparable initial body weights (47.2 ± 1.5 kg) as follows: (1) high AV and high β/AX (H_V_H_β/AX_); (2) high AV and medium β/AX (H_V_M_β/AX_); (3) high AV and low β/AX (H_V_L_β/AX_); (4) low AV and high β/AX (L_V_H_β/AX_); (5) low AV and medium β/AX (L_V_M_β/AX_); and (6) low AV and low β/AX (L_V_L_β/AX_).

### 2.3. Experimental Diet and Feeding Management

The methodologies for measuring and analyzing the nutritional components and physical characteristics of the feeds and diets followed our earlier works [19]. The specifics of the nutritional components, DF chemical components, and physical characteristics of the feeds are detailed in Table S1, while Table 1 presents the composition and nutritional content of the experimental diets. All experimental diets were formulated to maintain uniform nutrient content, adhering to the standardized ileal digestible crude protein, net energy, and total DF levels recommended by the National Research Council for growing pigs (NRC, 2012) [20]. The study was conducted over a 35-day period at the Pig Experiment Base of the Jilin Academy of Agricultural Sciences, beginning with a five-day adaptation period followed by 30 days of active experimentation. During the experiment, all pigs were housed in the same feeding trial room, with each pig individually kept in an independent pen of 1.8 m^2^. Each pen was equipped with a dedicated feeder and waterer (nipple drinker). The pens featured slatted floors with openings and semi-open partitions, facilitating visual communication and physical contact between adjacent pigs. The ambient temperature was maintained at 22.0 ± 1.0 °C, and the lighting period was approximately from 06:00 to 18:00 daily. Additionally, all pigs remained in good health throughout the experiment and did not receive any medications or vaccines. The feeding regimen involved administering diets at a daily intake that delivered triple the required energy for maintenance (419 kJ/kg body weight 0.75), and body weights were monitored weekly to adjust feed quantities accordingly [15,21]. During the experimental period, there were no restrictions on the animals’ water consumption, and they were fed twice daily, once at 08:00 AM and again at 04:00 PM, with each feeding being half of their daily ration.

### 2.4. Growth Performance

At the initiation and conclusion of the trial, we accurately recorded the pigs’ weight to ascertain their initial weight (IW) and final weight (FW), separately. This data facilitated the calculation of the average daily gain (ADG) throughout the trial. Concurrently, the dietary consumption of each pen was meticulously monitored, allowing for the documentation of the average daily feed intake (ADFI). To compute the feed-to-gain ratio (F/G), the total feed intake per pen was divided by the cumulative weight gain of the pigs housed within that pen.

### 2.5. Slaughtering Performance and Meat Quality Determination

Following the trial termination, the pigs were transported to the Jilin Zhengda Food Co., Ltd. Slaughterhouse (Changchun, China), where they were processed in alignment with established commercial standards and methodologies. Employing methods delineated in prior research [22,23], several key indicators were assessed and calculated, including lean meat percentage, loin-eye area, dressing percentage, carcass weight, fat percentage, back fat thickness, and abdominal fat thickness. These measurements served to evaluate the carcass characteristics comprehensively [24]. All experimental pigs were weighed 24 h prior to slaughter to ascertain their pre-slaughter live weights. The term “carcass weight” denotes the aggregate weight of the bilateral carcasses following exsanguination and depilation, excluding internal organs, tail, hooves, and head, but retaining the leaf fat and kidneys (while maintaining the leaf fat and kidneys). We measured the dressing percentage as carcass weight to pre-slaughter live weight ratio. The left side of each pig’s carcass was suspended in an inverted position, and the thickness of the fat was measured at three specified points along the dorsal midline: the thickest part, the last rib, and the lumbosacral junction, using a Vernier caliper. An average of these measurements was taken to determine the backfat thickness. In the case of the *Longissimus dorsi* muscle (LDM), it was dissected vertically at the position of the last rib on the left side. Sulfuric acid paper was placed over the cross-section, and the perimeter of the eye muscle was outlined with a dark pen to facilitate the measurement of the loin-eye area, which was subsequently quantified using a planimeter (Koizumi Sokki Co., Ltd., Tokyo, Japan). The weights of lean meat and fat were derived from standard formulas. We measured the lean meat weight as −6.9144 + 0.6154 × pre-slaughter live weight −2.6893 × backfat thickness at the 6th–7th ribs; the weight of fat was determined by Fat weight = −26.4 + 0.221 × carcass weight + 1.331. A portable colorimeter (MSEZ1900, Hunter Lab Corp., Reston, VA, USA) was employed to measure the color parameters—lightness (L*), redness (a*), and yellowness (b*)—of the LDM. Furthermore, a pH meter (S20, METTLER TOLEDO, Shanghai, China), calibrated with buffer solutions at pH 4.6 and 7, was utilized to determine the pH levels of each LDM sample at 45 min and 24 h post-mortem, with three replicates per sample. Muscle samples, each measuring 2 × 3 × 4 cm and replicated thrice, were excised from each pig 45 min post-slaughter for analyses of shear force, cooking loss, and drip loss. We determined the drip loss percentage by assessing the weight difference before and after suspension. Cooking loss was quantified by determining the percentage weight loss before and after cooking, relative to the initial weight. We used a texture analyzer (BROOKFIELD CT3 Texture Analyzer, Middleboro, MA, USA) fitted with a Warner-Bratzler shear force attachment for determining shear force. Marbling scores were assigned by three evaluators using the National Pork Producers Council standards for LDM samples refrigerated at 4 °C for 24 h. Finally, the dry matter (DM), crude protein (CP), and intramuscular fat (IMF) contents of the LDM were analyzed according to the protocols established by the Association of Analytical Chemists (AOAC 2007) [25].

### 2.6. Blood Biochemistry Analyses

On the morning of the 30th day of the formal experiment, researchers collected blood samples from the anterior vena cava of fasted pigs, which were then aliquoted into regular blood collection tubes (without anticoagulant for serum separation) and blood collection tubes containing the anticoagulant EDTA-K2 (for plasma separation). The collected blood was allowed to stand at ambient temperature for 30 min before being centrifuged at 1500× *g* for 15 min to separate plasma and serum. The samples were subsequently stored at −80 °C prior to analysis. An automated blood biochemical analyzer (BS-400, Mindray, Shenzhen, China) quantified several serum biochemical parameters, encompassing lactate dehydrogenase (LDH), triglycerides (TG), total cholesterol (TC), low-density lipoprotein cholesterol (LDL-C), high-density lipoprotein cholesterol (HDL-C), glucose (GLU), urea nitrogen (UN), albumin (ALB), and total protein (TP). Concentrations of glucagon-like peptide-1 (GLP-1) and peptide YY (PYY) in the plasma were determined using enzyme-linked immunosorbent assay kits (BIM Biosciences, Inc., San Francisco, CA, USA), under the producer’s guidance.

### 2.7. DNA Extraction, Library Construction, and Sequencing

The gut microbiota analysis included digesta samples from different intestinal segments of all experimental pigs (including the cecum, proximal colon, mid colon, distal colon, and rectum). Specifically, after opening the pigs’ abdominal cavities, the researchers ligated the start and end of the cecum, colon, and rectum before removing the entire large intestine and separating the cecum, colon, and rectum (sufficient ligation was performed to prevent digesta flow within the intestinal lumen). The colon was divided into three segments based on length, including the proximal colon (the side adjacent to the cecum), mid colon, and distal colon (the side adjacent to the rectum). The intestinal digesta samples of all pigs were collected according to the method described by Rattigan et al. and stored at −80 °C for microbiota determination [26]. We extracted total DNA from various intestinal digesta samples utilizing the E.Z.N.A.^®^ Soil DNA Kit (Omega Biotechnology, Norcross, GA, USA), following the instructions provided by the manufacturer. After successful quality control (QC) assessments, PCR amplification was conducted using primers 338F (5′-ACTCCTACGGGAGGCAGCA-3′) and 806R (5′-GGACTACHVGGGTWTCTAAT-3′). The PCR amplicons were subsequently purified using a DNA gel extraction and purification kit (PCR Clean-Up Kit, Yuhua, China) and quantified with a Qubit 4.0 fluorometer (Thermo Fisher Scientific, Waltham, MA, USA). Libraries were constructed using the NEXTFLEX Rapid DNA-Seq Kit (PerkinElmer, Waltham, MA, USA) and sequenced on an Illumina MiSeq PE300 platform (Illumina, San Diego, CA, USA). Following sequencing, QC and assembly of raw sequencing reads were performed using the DADA2 plugin within the Qiime2 pipeline (version 2019.4, Fort Lauderdale, FL, USA) to remove noise and generate amplicon sequence variants (ASVs). Taxonomic classification of the ASVs was conducted employing the Naive Bayes classifier in Qiime2 (version 2019.4, Fort Lauderdale, FL, USA), referencing the Silva 16S rRNA gene database (version 138). Data analysis was facilitated using the MajorBio Cloud Platform (MajorBio, Shanghai, China).

### 2.8. SCFA Concentration Analyses

The concentration of SCFA in the digesta was determined as per the methodology described by Tong et al. [27]. We weighed nearly 0.1 g of intestinal digesta, to which PBS solution was added at a 1:9 ratio. The mixture was vortexed for 2 min to ensure thorough mixing. This mixture was then centrifuged at 4500 rpm and 4 °C for 10 min. Subsequently, 300 μL of the resultant supernatant was transferred into a suitable container. To this, 25% metaphosphoric acid was added, maintaining a volume ratio of phosphoric acid to supernatant of 1:5, and the mixture was thoroughly vortexed. Following this, 250 μL of the mixture was withdrawn and co-extracted with an equivalent volume (250 μL) of ethyl acetate by vortexing. The supernatant was carefully aspirated with a syringe, filtered through an organic membrane (0.22 μm), and transferred into a gas-phase vial with an inner cannula. These vials were tightly sealed and stored at −20 °C until analysis. We used a gas chromatography system (Agilent Technologies 7890A-G3440A-GC System; Agilent Technologies, Santa Clara, CA, USA) for determining SCFA concentrations.

### 2.9. Quantitative Real-Time PCR Analysis

Total RNA was extracted using TRIzol (Takara Bio, Otsu, Japan) from the collected samples. The concentration of total RNA was quantified using a NanoDrop 2000 nucleic acid analyzer (Thermo Fisher, Waltham, MA, USA), and its purity was confirmed (OD260/280 between 1.9 and 2.0, indicating acceptable RNA purity). We used 1% agarose gel electrophoresis for evaluating RNA integrity. For reverse transcription, 1 μg of RNA was processed using a Prime Script^®^ RT Reagent Kit with gDNase (Takara Bio, Otsu, Japan). Primer sequences were designed based on the conserved regions of gene sequences retrieved from GenBank (National Center for Biotechnology Information, Bethesda, MD, USA) and were synthesized by Sangon Biotech Co., Ltd. (Shanghai, China). Primer efficiency was verified via standard curve analysis with 10-fold serial dilution of cDNA templates (5 gradients: 10^0^, 10^−1^, 10^−2^, 10^−3^, 10^−4^), and the efficiency values ranged from 95% to 105% with *R*^2^ > 0.99 for all primers, confirming specific and efficient amplification. A 20 μL reaction mixture, with gene-specific primers and SuperReal PreMix Plus (SYBR Green) Kit (Thermo Fisher, Waltham, MA, USA), was prepared and amplified using the ABI 7300 Real-Time PCR System (Applied Biosystems, Foster, CA, USA). Each sample was analyzed in triplicate. The relative expression levels were quantified using the cycle threshold (Ct) method, with β-actin as the single reference gene. β-actin was selected for normalization due to its stable expression across all experimental groups (Ct value variation < 0.5 among samples). The specific primer sequences are listed in Table S2.

### 2.10. Statistical Analysis

The statistical model employed was *Y_ijk_* = *µ* + *α_i_* + *β_j_* + *α_i_* × *β_j_* + *ɛ_ijk_*, where *µ* represents the population mean, *α_i_* signifies the effect of dietary AV (*i* = 1, 2), *β_j_* denotes the levels of β/AX (*j* = 1, 2, 3), *α_i_* × *β_j_* represents the interaction between dietary AV and β/AX, and *ɛ_ijk_* reflects the residual variance. Data were analyzed using SPSS version 27.0 (IBM Corp., Armonk, NY, USA). The assumptions of normality and homogeneity of variance were tested using Levene’s test. A two-way ANOVA was conducted to assess the effects of dietary AV, β/AX, and their interaction. Differences among the six dietary treatments were evaluated using Tukey’s multiple comparison test. Bar charts depicting the results were generated with GraphPad Prism version 10 (GraphPad Software, San Diego, CA, USA). Correlation patterns were analyzed using Pearson’s correlation coefficient and visualized as a heatmap with Origin 2021 software (Origin Lab Corporation, Northampton, MA, USA). Microbial composition and diversity were analyzed on the MajorBio Microbial Cloud platform (MajorBio, Shanghai, China). Statistical significance was noted at *p*-values below 0.01 (highly significant), below 0.05 (significant), and between 0.05 and 0.1 (indicative of a trend). Results are presented as mean ± standard error.

## 3. Results

### 3.1. Adjusting the Dietary β/AX and AV Alters the Weight Gain and Feed Conversion Efficiency of Pigs

The study investigates the effects of alterations in the chemical structure and AV of DF on the growth performance of pigs, provided that the concentrations of other nutrients (energy, protein, and DF) remain constant. The analysis, summarized in Table 2, revealed a notable linear interaction between dietary β/AX and AV on two critical growth metrics: ADG and F/G (*p* < 0.05). In comparison to the group with low dietary AV (L_V_), the group with high dietary AV (H_V_) exhibited decreases in FW and ADG (*p* < 0.001) and an increase in the F/G ratio (*p* < 0.001). Similarly, an elevation in dietary β/AX led to reductions in FW and ADG (*p* < 0.05) and an increase in the F/G ratio (*p* < 0.001), though it did not notably alter the ADFI (*p* > 0.05). An examination across six dietary treatment groups demonstrated that both dietary β/AX and AV notably impacted FW, ADG, and F/G ratio (*p* < 0.05). Furthermore, dietary β/AX exhibited a pronounced first-order linear effect on these growth parameters (*p* < 0.001).

### 3.2. Increasing the Dietary β/AX and AV Can Reduce Fat Deposition in Pigs

The impact of the chemical structure and AV of DF on the carcass characteristics of pigs is detailed in Table 3. Increases in dietary β/AX and AV correlated with reductions in pre-slaughter live weight, carcass weight, and dressing percentage (*p* < 0.05), consistent with the trends observed in growth performance. There was a notable interaction effect between dietary β/AX and AV on both carcass weight and dressing percentage (*p* < 0.001). Notably, higher levels of dietary β/AX and AV contributed to reductions in fat percentage, fat weight, backfat thickness, and abdominal fat thickness, presenting a notable interaction (*p* < 0.05), while showing no notable effects on the loin-eye area and lean meat weight (*p* > 0.05). Additionally, the percentage of lean meat exhibited an increasing trend with higher dietary β/AX and AV (0.10 > *p* ≥ 0.05). A comparative analysis among the six dietary treatment groups indicated notable impacts of dietary β/AX and AV on pre-slaughter live weight, carcass weight, dressing percentage, fat percentage, fat weight, backfat thickness, and abdominal fat thickness (*p* < 0.05), with these effects being linearly influenced by dietary β/AX (*p* < 0.05).

### 3.3. Increasing the Dietary β/AX and AV Improves the Tenderness of Meat

Table 4 delineates the impact of DF structure and AV on meat quality. The analysis of physical characteristics revealed an interaction effect between dietary β/AX ratios and AV on shear force (*p* < 0.05). However, no notable interaction was observed for other quality indicators (*p* > 0.05). Notably, the shear force in the H_V_ group was notably reduced compared to the L_V_ group (*p* < 0.05). Among the β/AX ratio groups, the high β/AX ratio (H_β/AX_) group exhibited a notably lower shear force than the low β/AX ratio (L_β/AX_) group (*p* < 0.05). The medium β/AX ratio (M_β/AX_) group also displayed a trend toward reduced shear force relative to the L_β/AX_ group, although this difference did not reach statistical significance (*p* > 0.05). Increasing dietary AV was associated with a significant elevation in the a*_24 h_ value of meat (*p* < 0.05) and showed a trend toward an increased b*_24 h_ value (*p* = 0.061). Moreover, the chemical composition of the meat was notably influenced by the dietary β/AX and AV, particularly affecting the DM content of LDM. An interaction effect was evident (*p* < 0.001), with the DM content in the LDM being higher in the H_V_ group than in the L_V_ group (*p* < 0.05). Similarly, the DM content in the H_β/AX_ group was elevated compared to that in the L_β/AX_ group (*p* < 0.05), although there was no significant variance when compared with the M_β/AX_ group (*p* > 0.05). Additionally, increasing both dietary β/AX and AV demonstrated a trend toward higher CP and IMF content in the LDM, although these differences did not achieve statistical significance (*p* > 0.05).

### 3.4. Dietary β/AX and AV Affect the Body’s Lipid Metabolism

Table 5 delineates the impact of DF structure and AX on the serum biochemical indices in pigs. The findings indicate a linear interactive effect of dietary β/AX ratios and AV on the concentrations of fasting blood GLU, TC, and HDL-C in pigs (*p* < 0.05). An increase in the AV of the diet was associated with reduced concentrations of GLU, TC, and TG in pig serum (*p* < 0.05), whereas the concentrations of TP, UN, LDL-C, ALB, and LDH remained unaffected (*p* > 0.05). Similarly, an increase in dietary β/AX ratios linearly decreased the concentrations of serum GLU and TC (*p* < 0.05) and elevated HDL-C levels (*p* < 0.05). Additionally, increasing dietary β/AX ratios exhibited a tendency to reduce serum LDL-C levels (*p* = 0.057), though this reduction did not reach statistical significance (*p* > 0.05). Moreover, variations in dietary β/AX ratios and AV also influenced plasma concentrations of GLP-1 and PYY (Figure S1). There were significant interaction effects between dietary β/AX ratios and AV on plasma GLP-1 levels in pigs (*p* < 0.05). An increase in dietary AV elevated plasma GLP-1 concentrations (*p* < 0.05) but did not notably impact PYY concentrations (*p* > 0.05). An enhancement in dietary β/AX ratios also increased plasma GLP-1 levels (*p* < 0.05) and showed a tendency to raise PYY levels (*p* = 0.054).

### 3.5. The Dietary β/AX and AV Regulate the Expression of Genes Related to Lipid Metabolism in Liver

**Figure 1:**
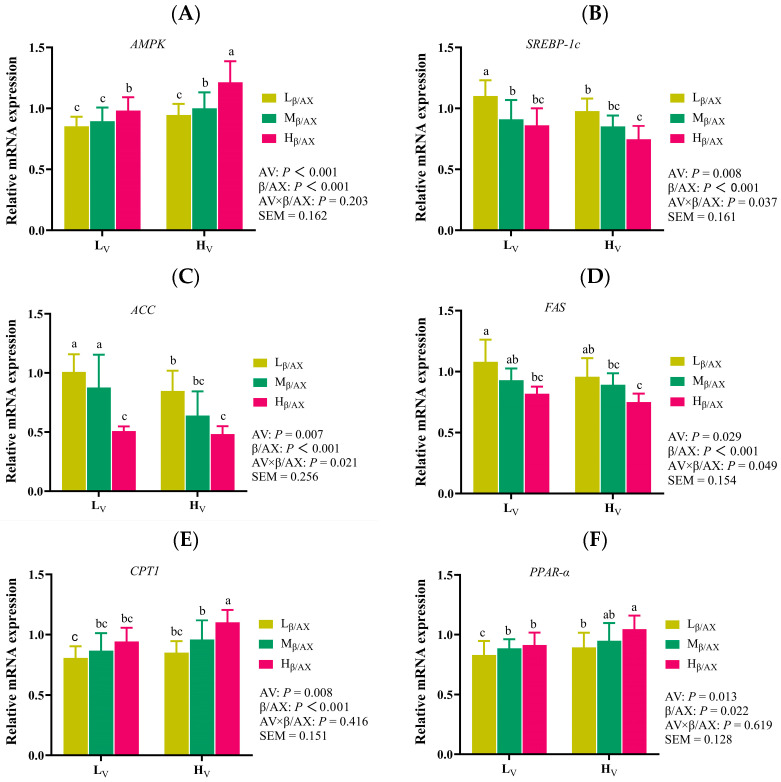
Effects of dietary fiber chemical structure and apparent viscosity on the expression of genes related to liver lipid metabolism. The bar chart presents the differential analysis of the expression levels of lipid metabolism-related genes such as *AMPK* (**A**), *SREBP-1C* (**B**), *ACC* (**C**), *FAS* (**D**), *CPT1* (**E**), and *PPAR-α* (**F**) in the liver. Data have an expression of Mean ± SD and significance is indicated: distinct lowercase letters are indicative of notable discrepancies (*p* < 0.05). AV: apparent viscosity; β/AX: β-glucan-to-arabinoxylan ratio; AV × β/AX: interaction effect between the dietary apparent viscosity and β-glucan-to-arabinoxylan ratio; AMPK: AMP-activated protein kinase; SREBP-1C: sterol regulatory element-binding protein-1C; ACC: acetyl-CoA carboxylase; FAS: fatty acid synthase; CPT1: carnitine palmitoyltransferase 1; PPAR-α: peroxisome proliferator-activated receptor alpha; L_V_: low apparent viscosity; H_V_: high apparent viscosity; L_β/AX_: low β-glucan-to-arabinoxylan ratio; M_β/AX_: meddle β-glucan-to-arabinoxylan ratio; H_β/AX_: high β-glucan-to-arabinoxylan ratio; SEM: standard error of the mean.

To further investigate the potential mechanisms through which the structure and physical characteristics of DF modulate subcutaneous fat deposition and lipid metabolism in pigs, we analyzed the mRNA expression levels of key lipid metabolism genes in the liver. As depicted in Figure 1, a significant interaction effect was observed between dietary β/AX ratios and AV on the mRNA expression levels of sterol regulatory element-binding protein-1C (*SREBP-1C*), acetyl-CoA carboxylase (*ACC*), and fatty acid synthase (*FAS*) in the liver (*p* < 0.05). An increase in the AV of the diet enhanced the mRNA expression of AMP-activated protein kinase (*AMPK*), carnitine palmitoyltransferase 1 (*CPT1*), and peroxisome proliferator-activated receptor alpha (*PPAR-α*) (*p* < 0.05) and concurrently decreased the expression of *SREBP-1C*, *ACC*, and *FAS* (*p* < 0.05). Likewise, an elevation in dietary β/AX ratios augmented the mRNA levels of *AMPK*, *CPT1*, and *PPAR-α* (*p* < 0.05) and notably reduced the expression of *SREBP-1C*, *ACC*, and *FAS* (*p* < 0.001).

### 3.6. The Dietary β/AX and AV Selectively Regulate the Microbial Composition in the Middle Colon

**Figure 2:**
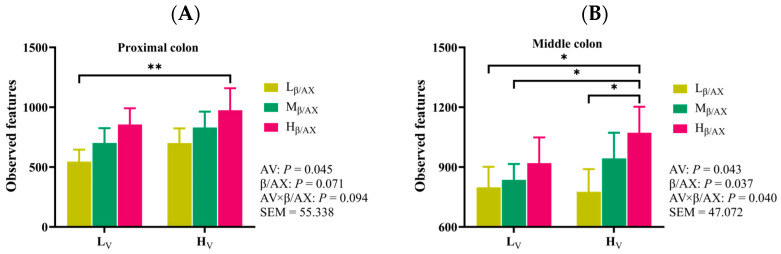

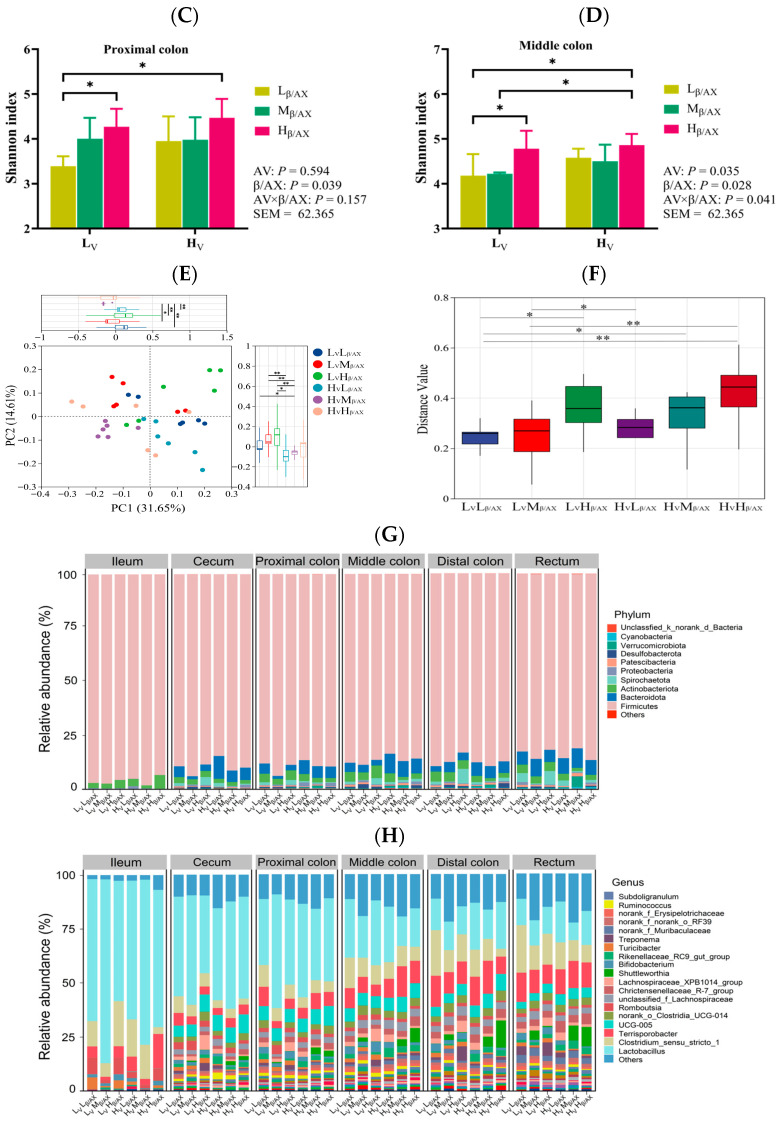
Effects of the chemical structure and physical characteristics of DF on the diversity and composition of gut microbiota. The bar chart show the number of observed microbial species in the proximal colon (**A**) and in the middle colon (**B**), as well as the Shannon index of microorganisms in the proximal colon (**C**) and in the middle colon (**D**). The (**E**) shows the β-diversity analysis of middle colon microorganisms. The box plots show the inter-group difference analysis of the β-diversity index (**F**). The stacked column chart shows the microbial composition at the phylum (**G**) and genus (**H**) levels. AV: apparent viscosity; β/AX: β-glucan-to-arabinoxylan ratio; AV × β/AX: interaction effect between the dietary apparent viscosity and β-glucan-to-arabinoxylan ratio; L_V_: low apparent viscosity; H_V_: high apparent viscosity; L_β/AX_: low β-glucan-to-arabinoxylan ratio; M_β/AX_: meddle β-glucan-to-arabinoxylan ratio; H_β/AX_: high β-glucan-to-arabinoxylan ratio; SEM: standard error of the mean; L_V_L_β/AX_: diet with low apparent viscosity and low β-glucan-to-arabinoxylan ratio; L_V_M_β/AX_: diet with low apparent viscosity and middle β-glucan-to-arabinoxylan ratio; L_V_H_β/AX_: diet with low apparent viscosity and high β-glucan-to-arabinoxylan ratio; H_V_L_β/AX_: diet with high apparent viscosity and low β-glucan-to-arabinoxylan ratio; H_V_M_β/AX_: diet with high apparent viscosity and middle β-glucan-to-arabinoxylan ratio; H_V_H_β/AX_: diet with high apparent viscosity and high β-glucan-to-arabinoxylan ratio. * *p* < 0.05, ** *p* < 0.01.

16S rRNA amplicon sequencing was utilized to delineate the influence of DF’s physicochemical characteristics on the gut microbiota across various gastrointestinal regions, from the ileum to the large intestine in pigs. Analysis of α-diversity across different dietary treatment groups revealed marked differences in microbial diversity within the proximal and middle colons, evidenced by variations in the number of observed species and the Shannon index (Figure 2A–D and Figure S2). In contrast, variations in the ileum and other segments of the large intestine were comparatively minor. Notably, there was a correlation between enhanced dietary β/AX and AV levels and increased α-diversity of microorganisms. Subsequent β-diversity analysis of the microbial community structure, employing principal coordinate analysis (PCoA) and inter-group difference analysis, indicated a significant modulation of the microbial community within the middle colon by dietary β/AX and AV (Figure 2E,F). The structure of the microbial community displayed distinct segregation, with significant disparities among the various dietary groups. This pattern underscores the spatial specificity in the regulation of pig gut microbiota by the physicochemical attributes of DF, with the middle colon being the primary site of influence. Further taxonomic breakdown identified 19 phyla, 153 families, 357 genera, 759 species, and 78,763 ASVs. The predominant phyla and genera included Firmicutes, Bacteroidota, Actinobacteriota, Spirochaetota, Proteobacteria, and the genera *Lactobacillus*, *Clostridium_sensu_stricto_1*, *Terrisporobacter*, *UCG-005*, and *Clostridia_UCG-014* (Figure 2G,H). Within the ileum, *Lactobacillus* was the dominant genus. However, as the gastrointestinal tract extended posteriorly towards the large intestine, the microbial composition diversified notably, with a gradual replacement of *Lactobacillus* by other genera and no single genus dominating. Analysis of genus-level abundance variations revealed that an increase in dietary AV was associated with higher abundances of *Terrisporobacter*, *Rikenellaceae_RC9*, and *Muribaculaceae*, whereas it led to a decrease in *Bifidobacterium* and *Romboutsia* abundances (Figure S3; *p* < 0.01). Additionally, there was a trend towards an increased abundance of *Shuttleworthia* (*p* = 0.070). Similarly, enhanced dietary β/AX levels were correlated with increased abundance of *Rikenellaceae_RC9* and *Muribaculaceae* and a decreased relative abundance of *Bifidobacterium* (*p* < 0.01). Notably, an interaction effect between dietary β/AX and AV was observed impacting the changes in *Terrisporobacter* in the middle colon (*p* < 0.05).

### 3.7. Dietary β/AX and AV Enhance the Production of Butyrate in the Middle Colon

To explore the influence of DF’s structural and physical characteristics on gut microbiota metabolites, alterations in the concentrations of SCFA within the middle colon were monitored. As indicated in Table 6, an increase in the AV of the diet was associated with elevated levels of acetate, butyrate, and total short-chain fatty acids (TSCFA) in the middle colon of pigs (*p* < 0.05). Concurrently, a rise in dietary AV tended to increase propionate production and decrease isobutyrate production, although these changes were not statistically significant (*p* > 0.05). Moreover, an increase in the dietary β/AX was linked to heightened production of acetate, butyrate, valerate, and TSCFA (*p* < 0.05), demonstrating a linear relationship with butyrate and TSCFA production (*p* < 0.05). Notably, a linear interaction effect between dietary β/AX and AV on the production of butyrate and TSCFA was observed in the middle colon (*p* < 0.05).

### 3.8. The Dominant Bacterial Genera Regulated by the Dietary β/AX and AV Are Related to the Production of Butyrate

**Figure 3:**
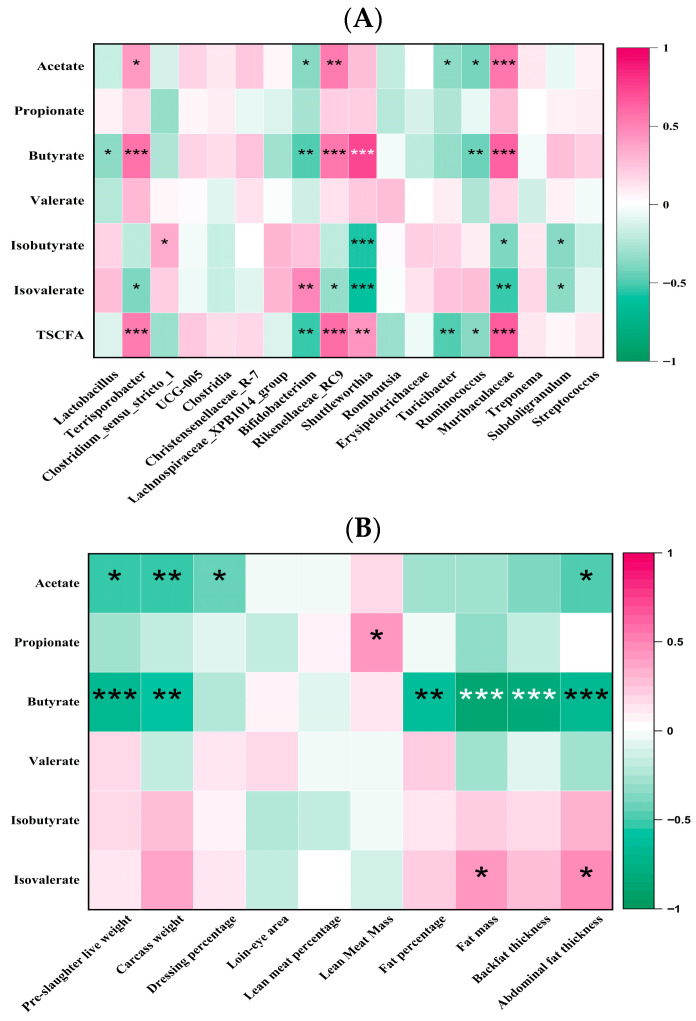
Correlation analysis between short-chain fatty acids and gut microbiota (**A**) as well as carcass traits (**B**). The heatmap of the correlation matrix shows Spearman’s correlation coefficients. TSCFA: total short-chain fatty acids. Deep green suggests strong negative associations, and colors close to deep red indicate strong positive associations. * *p* < 0.05, ** *p* < 0.01, *** *p* < 0.001.

Correlation analyses between the microbial genera in the middle colon and SCFA concentrations revealed that acetate production was positively associated with the genera *Terrisporobacter*, *Rikenellaceae_RC9*, and *Muribaculaceae* (*R* > 0.41, *p* < 0.05) and inversely correlated with the relative abundances of *Bifidobacterium*, *Turicibacter*, and *Ruminococcus* (Figure 3A; *R* < −0.34, *p* < 0.05). Butyrate production showed strong positive correlations with *Terrisporobacter*, *Rikenellaceae_RC9*, *Shuttleworthia*, and *Muribaculaceae* (*R* > 0.57, *p* < 0.001), and negative correlations with genera such as *Lactobacillus*, *Bifidobacterium*, and *Ruminococcus* (*R* < −0.35, *p* < 0.05). No clear correlation was established between propionate production and microbial genera alterations. Additionally, *Shuttleworthia*, *Muribaculaceae*, and *Subdoligranulum* exhibited negative correlations with the production of isobutyrate and isovalerate (*R* < −0.35, *p* < 0.05). The production of TSCFA was positively correlated with *Terrisporobacter*, *Rikenellaceae_RC9*, *Shuttleworthia*, and *Muribaculaceae* (*R* > 0.43, *p* < 0.01), and negatively with genera such as *Bifidobacterium*, *Turicibacter*, and *Ruminococcus* (*R* < −0.37, *p* < 0.05).

### 3.9. The Increase in Butyrate Is Associated with the Reduction in Fat Deposition

The results of the Pearson correlation analysis between SCFA in the middle colonic digesta and various carcass traits are depicted in Figure 3B. Notably, an elevation in butyrate levels, influenced by increased dietary β/AX and AV, exhibits a strong association with fat accumulation and body weight alterations. Specifically, butyrate production demonstrated a significant negative correlation with several metrics, including fat mass, backfat thickness, abdominal fat thickness, fat percentage, pre-slaughter live weight, and carcass weight (*R* < 0.55, *p* < 0.01). Similarly, acetic acid production displayed significant negative correlations with abdominal fat thickness, pre-slaughter live weight, carcass weight, and slaughter rate (*R* < 0.42, *p* < 0.05). Conversely, propionate showed a significant positive correlation with lean meat weight (*R* > 0.41, *p* < 0.05), and isovalerate was positively correlated with both fat mass and abdominal fat thickness (*R* > 0.40, *p* < 0.05).

## 4. Discussion

Extant research underscores the pivotal role that DF and its microbial fermentation metabolites play in lipid metabolism regulation [28,29,30]. Previous investigations have predominantly concentrated on the quantity or source of DF, or on isolated fiber components and their physicochemical characteristics, to elucidate their beneficial impacts on pigs and other species [5,31,32,33,34,35]. However, the use of synthetic or purified fibers, which constitute single components within the broader spectrum of DF, does not adequately mirror the nutritional characteristics and functional attributes of naturally derived plant fibers. Often, these isolated fibers do not fully capture the comprehensive effects of DF on pig nutrition and metabolism [19,36,37,38]. In this study, we explore the regulatory effects and potential mechanisms through which the physicochemical characteristics (chemical structure and physical characteristics) of natural DFs influence pig growth performance, carcass traits, meat quality, and gut microbiota composition. Our preliminary findings suggest that dietary β/AX and AV synergistically influence subcutaneous fat deposition and improve certain meat quality characteristics (*p* < 0.05). We observed that modifications in dietary β/AX and AV could regulate serum lipid metabolism indicators and the expression of crucial genes involved in lipid metabolism within the liver. Moreover, microbial sequencing across various intestinal locations indicated that dietary β/AX and AV predominantly affect the microbiome in the middle colon and modulate the abundance of butyrate-producing bacterial genera. These results unveil the potential mechanisms through which the gut-liver axis is influenced by the physicochemical characteristics of DF, thereby regulating subcutaneous fat deposition in pigs.

Numerous studies have demonstrated that excessive lipid accumulation presents significant health risks for both humans and animals [39,40,41]. In the realm of animal production, excessive fat deposition is recognized as a critical factor that adversely affects fattening efficiency, meat quality, reproductive capacity, and immune performance [42,43]. DF, a vital component of pig diets, interacts complexly with other nutrients, influencing nutrient digestion and metabolism. Furthermore, it acts as a crucial substrate for gut microbes, undergoing fermentation and degradation to yield metabolites such as SCFA, which contribute to the host’s energy metabolism. In our preceding studies, we elucidated that augmenting dietary β/AX and AV levels diminishes the apparent ileal digestibility of DM and organic matter in pigs, thereby enhancing the excretion of fecal and urinary energy [19]. Corroborating these findings, the current study reveals that diets enriched with higher β/AX and AV concentrations depress ADG in pigs, subsequently reducing carcass weight and slaughter yield. Intriguingly, while elevated levels of dietary β/AX and AV compromise slaughter performance, they do not detract from the proportion of lean meat. On the contrary, there appears to be an increase in the yield of lean meat per unit body weight. Notably, the augmentation in dietary β/AX and AV markedly curtails subcutaneous fat deposition. These results imply that although modifications in the chemical structure and physical characteristics of DF somewhat impede weight gain efficiency, they concurrently enhance carcass quality and fattening efficiency. Parallel findings were reported by Lu et al. [29], who observed that raising the dietary neutral detergent fiber content to 16% diminished the pre-slaughter live weight and carcass weight of pigs, as well as the carcass fat content (backfat thickness and fat percentage), while increasing the loin eye area and net lean meat percentage, and ameliorating meat tenderness. These outcomes resonate with those of the present study, which showed that tweaking the chemical structure and physical characteristics of fiber, within a consistent DF level, ameliorates the tenderness and nutritional composition of pork. Additionally, Li et al. [44] reported analogous effects. Their research indicated that increasing the proportion of ramie fiber in the diet reduced growth performance and slaughter traits such as ADG, carcass weight, and backfat thickness, yet improved meat tenderness and increased protein content in the meat. These collective findings underscore the multifaceted regulatory effects of DF on the growth performance, carcass traits, and meat quality of pigs, influenced not only by its level and source but also by the interaction between its chemical structure and physical characteristics. Additionally, based on the results of this study, reducing dietary β/AX and AV is more beneficial to the weight gain and feed conversion rate of growing pigs (though the differences in dietary β/AX and AV are mainly reflected in subcutaneous fat deposition). Therefore, when growing pigs are fed high-fiber diets or diets supplemented with more agricultural by-products, the effects of dietary fiber structure and apparent viscosity (including interaction effects and independent effects) should be considered. However, appropriately increasing dietary β/AX and AV may be more favorable for the feeding strategy of finishing pigs to improve their finishing efficiency and meat quality.

In recent years, a substantial body of research has been devoted to elucidating the intricate relationships between the gut microbiomes of humans and animals and their associations with various diseases, in addition to their significant roles in nutrition, metabolism, physiology, and immune function [45,46]. It is now well-established that gut microbes play a pivotal role in regulating the host’s fat deposition phenotype, nutrient utilization efficiency, and production performance [47,48]. Building on these findings, our study explores the mechanisms by which dietary β/AX and AV influence fat deposition in pigs, mediated by the gut-liver axis effect, which is contingent upon the physicochemical characteristics of DF. Through the analysis of alpha and beta diversities across different groups, our research has identified significant variations in the microbial community structures across various segments of the digestive tract, including the ileum, cecum, proximal colon, middle colon, distal colon, and rectum. Notably, the microbial communities in the middle colon are crucially influenced by the chemical structure and physical characteristics of the DF. Our findings indicate that adjusting the levels of dietary β/AX and AV selectively enhances the relative abundance of acid-producing bacterial genera such as *Terrisporobacter*, *Rikenellaceae_RC9*, *Shuttleworthia*, and *Muribaculaceae* in the middle colon. These bacterial genera have been linked to obesity in prior research, suggesting potential interventions and associations [49,50,51]. Furthermore, Jiang et al. [33] demonstrated that dietary supplementation with β-glucan hydrogels, which are highly water-absorbent, mitigated obesity in mice on a high-fat diet. Their study also revealed a negative correlation between obesity-related traits and the *Muribaculaceae* genus. Additionally, our correlation analysis indicates that these bacterial genera, which are upregulated by increased levels of dietary β/AX and AV, are positively associated with the production of butyrate in the middle colon, echoing findings from previous studies [51,52]. These outcomes suggest that, under consistent dietary nutrient conditions, altering dietary β/AX and AV effectively modulates the proportion of butyrate-producing bacterial genera in the middle colon of pigs and enhances butyrate production.

The significance of SCFA, particularly butyric acid, in linking microbial activities to diverse biological effects and phenotypes has been well-documented [53,54]. SCFA serve as vital energy sources for the colonic epithelium. Studies have shown that dietary supplementation with butyric acid enhances energy expenditure and fat oxidation in obese mice, effectively preventing obesity triggered by a high-fat diet [55]. Furthermore, Zhou et al. [56] demonstrated that supplementation with a combination of acetic acid, propionic acid, and butyric acid reduces fat deposition in pigs, regardless of the involvement of the gut microbiota. In a recent investigation, the augmentation of β/AX and AV in pig diets was found to synergistically enhance butyrate production, which in turn increased the absorption of butyric acid, as evidenced by a significant elevation in butyrate concentrations in the hepatic portal vein plasma [19]. Additionally, butyrate production in the middle colon exhibited a strong inverse correlation with several indicators of fat deposition and weight gain, including fat mass, backfat thickness, abdominal fat thickness, fat percentage, pre-slaughter live weight, and carcass weight. These findings suggest that the regulatory effects of dietary β/AX and AV on fat deposition may be mediated through butyrate. Previous research has identified the anti-obesity effects of butyrate as being facilitated by the *AMPK-ACC* pathway, which diminishes fat deposition [56], aligning with the outcomes observed in our study. We noted that an increase in dietary β/AX and AV led to elevated mRNA expression of *AMPK* and *PPAR-α* in the liver, alongside enhanced activity of *CPT-1*. Conversely, mRNA expression of *SREBP-1C* in the liver, as well as the activities of *FAS* and *ACC*, were notably reduced in the group receiving high dietary β/AX and AV. These results corroborate previous findings in pig liver, muscle, and adipose tissues [57,58,59]. *AMPK*, recognized as a critical energy sensor, governs cellular metabolism and influences lipid metabolic homeostasis [60]. Research indicates that activation of *AMPK* not only promotes energy catabolism, such as fatty acid oxidative catabolism, but also inhibits anabolic processes, including the de novo synthesis of fatty acids [61]. This dual role establishes *AMPK* as a central regulator of lipid synthesis and oxidation.

Additionally, *SREBP-1C* and *PPAR-α* are pivotal transcription factors that govern the de novo synthesis of fat and the β-oxidation catabolism pathway of fatty acids, separately [62]. The principal downstream target genes include *FAS*, *ACC*, and *CPT-1*, which play crucial roles in lipid synthesis and metabolism [63]. Activation of *AMPK* typically inhibits the *SREBP-1C* pathway while simultaneously enhancing the PPAR-α signaling pathway [62,63]. Consequently, the proposed mechanism by which dietary modification with β/AX and AV reduces fat deposition in pigs in this study may involve butyric acid, which, mediated by the chemical structure and physical characteristics of fiber, activates the *AMPK-SREBP-1C* pathway and diminishes the activities of *FAS* and *ACC*, thus curtailing de novo fat synthesis. Conversely, through the *AMPK-PPAR-α* pathway, butyric acid decreases ACC activity and augments the expression of *CPT-1*, thereby fostering the oxidation of fatty acids [64]. These effects align with the observed outcomes of serum lipid metabolism indicators. Furthermore, an increase in dietary β/AX and AV was found to decrease serum glucose concentrations, exhibiting an interaction effect. This effect likely stems from alterations in the chemical structure and physical characteristics of DF, which impact the rheological properties and the rate of gastric emptying. Recent studies on the viscosity characteristics of fiber in porcine nutrition have demonstrated that the inclusion of purified β-glucan in the diet elevates the viscosity of intestinal digesta [15]. Such an increase in viscosity may attenuate the interaction between digestive enzymes and the chyme matrix and engage with the intestinal mucosa to create an absorption barrier, thus reducing the uptake of specific nutrients. Moreover, an enhanced dietary content of β/AX and AV has been associated with increased plasma GLP-1 concentrations, further underscoring the significant role of the chemical and physical characteristics of DF in modulating glucose and lipid metabolism in pigs.

These findings elucidate the intricate interplay between the physicochemical characteristics of DF and host fat deposition mechanisms, including lipid synthesis and metabolism. This evidence offers fresh perspectives on how to optimize fattening efficiency and meat quality in pigs through tailored fiber nutrition and illustrates potential strategies for utilizing DF effectively to manage obesity and prevent the onset of nutritional and metabolic disorders. Further research in this field may reveal the overall effects of DF intervention on lipid metabolism and regulation of animal production performance and welfare, and provide a basis for the feeding strategies of high-fiber diets and the effective utilization of agricultural by-products in pig production. However, this study has limitations such as a relatively short experimental period and a small number of experimental animals. Extending the experimental period will help further understand the overall effects of differences in DF structure and AV on the production performance and product quality of pigs at different physiological stages (growing and finishing phases). In addition, subsequent studies should examine whether the potential mechanisms by which DF structure and AV regulate fat deposition and production performance in pigs are associated with changes in digesta transit kinetics and substrate fermentation kinetics in the large intestine. Therefore, it is necessary to increase the number of experimental animals and extend the experimental period, and combine cutting-edge technologies such as metagenomics, metabolomics, and lipidomics to verify and expand these findings.

## 5. Conclusions

In summary, our research demonstrates the interaction effect of dietary DF’s chemical structure and physical properties in regulating fat deposition and meat quality in pigs. With consistent DF intake and nutrient provision, an elevation in dietary β/AX ratio and AV was found to influence lipid synthesis and oxidation pathways via the butyrate-mediated gut-liver axis, thereby modulating subcutaneous fat deposition in pigs. Reducing the β/AX and AV in high DF diets can improve the growth performance of pigs. This study provides new insights into the application strategy of unconventional feeds in pig production.

## Figures and Tables

**Table 1:** Ingredients and chemical composition of experimental diets ^a^ (%, as-fed basis).

▪ Items	L_V_L_β/AX_	L_V_M_β/AX_	L_V_H_β/AX_	H_V_L_β/AX_	H_V_M_β/AX_	H_V_H_β/AX_
▪ Ingredients						
▪ Corn	29.00	18.70	8.90	-	-	-
▪ Barley	20.00	29.50	39.00	24.00	35.00	46.00
▪ Wheat	8.00	10.00	12.00	38.96	28.44	17.96
▪ Soybean meal	3.00	2.35	1.70	2.00	3.60	5.20
▪ Rapeseed meal	-	-	-	-	4.00	8.00
▪ Corn gluten meal	3.00	1.75	-	4.00	2.00	-
▪ Corn DDGS	5.00	11.75	18.50	-	-	-
▪ Corn husk	9.82	9.90	10.00	-	-	-
▪ Soybean husk	12.00	6.00	-	4.00	2.00	-
▪ Sugar beet pulp	-	-	-	16.00	14.00	12.00
▪ Soybean oil	4.80	4.65	4.50	5.20	5.35	5.50
▪ Salt	0.80	0.80	0.80	0.80	0.80	0.80
▪ Limestone	1.50	1.65	1.80	1.50	1.50	1.50
▪ Dicalcium phosphate	0.40	0.41	0.42	0.80	0.61	0.42
▪ L-Lysine (98%)	0.77	0.75	0.73	0.75	0.66	0.57
▪ L-Methionine (99%)	0.12	0.13	0.14	0.14	0.15	0.15
▪ L-Cysteine (99%)	0.03	0.04	0.04	0.03	0.03	0.03
▪ L-Threonine (98%)	0.31	0.31	0.31	0.33	0.33	0.32
▪ L-Tryptophan (98%)	0.09	0.08	0.07	0.06	0.05	0.04
▪ L-Isoleucine (99%)	0.11	0.09	0.06	0.07	0.06	0.05
▪ L-Leucine (99%)	0.04	0.02	-	0.20	0.28	0.35
▪ L-Valine (99%)	0.12	0.07	0.02	0.10	0.08	0.06
▪ L-Histidine (99%)	0.03	0.02	0.01	0.06	0.05	0.04
▪ L-Phenylalanine (99%)	0.06	0.03	-	-	0.01	0.01
▪ TiO_2_	0.40	0.40	0.40	0.40	0.40	0.40
▪ Vitamin and mineral premix ^b^	0.60	0.60	0.60	0.60	0.60	0.60
▪ Total	100.00	100.00	100.00	100.00	100.00	100.00
▪ Calculated nutrition content ^c^ (%, as DM)						
▪ Net energy (MJ/kg)	10.42	10.46	10.49	10.50	10.51	10.51
▪ Crude protein (SID) ^d^	10.82	10.83	10.84	10.84	10.83	10.83
▪ Nitrogen-free extract	54.13	54.91	55.69	54.51	54.40	54.28
▪ Ash	2.25	2.35	2.44	2.40	2.66	2.93
▪ Total dietary fiber	23.01	22.78	22.56	23.28	23.26	23.24
▪ β-glucan	2.05	3.08	4.11	2.31	2.87	3.52
▪ Arabinoxylan	8.58	8.95	9.08	8.91	8.07	7.71
▪ β-glucan-to-arabinoxylan ratios	0.24	0.34	0.45	0.26	0.36	0.46
▪ Apparent viscosity (cP)	1.04	1.05	1.05	1.21	1.21	1.21
▪ Calcium	0.73	0.73	0.74	0.75	0.74	0.74
▪ Available phosphorus	0.24	0.24	0.25	0.24	0.25	0.24
▪ Lysine (SID)	0.97	0.96	0.96	0.97	0.97	0.98
▪ Methionine (SID)	0.30	0.30	0.30	0.30	0.30	0.30
▪ Cysteine (SID)	0.26	0.26	0.26	0.26	0.26	0.26
▪ Threonine (SID)	0.60	0.60	0.59	0.60	0.60	0.60
▪ Tryptophan (SID)	0.17	0.17	0.16	0.17	0.17	0.17
▪ Leucine (SID)	1.21	1.22	1.23	1.22	1.22	1.22
▪ Valine (SID)	0.65	0.65	0.64	0.65	0.65	0.65
▪ Arginine (SID)	0.52	0.54	0.55	0.52	0.60	0.67
▪ Histidine (SID)	0.33	0.33	0.33	0.33	0.33	0.33
▪ Phenylalanine (SID)	0.60	0.61	0.62	0.61	0.61	0.60

^a^ L_V_L_β/AX_: diet with low apparent viscosity and low β-glucan-to-arabinoxylan ratio; L_V_M_β/AX_: diet with low apparent viscosity and middle β-glucan-to-arabinoxylan ratio; L_V_H_β/AX_: diet with low apparent viscosity and high β-glucan-to-arabinoxylan ratio; H_V_L_β/AX_: diet with high apparent viscosity and low β-glucan-to-arabinoxylan ratio; H_V_M_β/AX_: diet with high apparent viscosity and middle β-glucan-to-arabinoxylan ratio; H_V_H_β/AX_: diet with high apparent viscosity and high β-glucan-to-arabinoxylan ratio; Corn DDGS: corn distillers dried grains with solubles. ^b^ Supplied the following per kilogram complete diet, vitamin A, 28,500 IU; vitamin D, 36,000 IU; vitamin E, 67.5 IU; vitamin K, 37.5 mg; vitamin B_1_, 17.5 mg; vitamin B_2_, 215 mg; vitamin B_6_, 69 mg; vitamin B_12_, 0.075 mg, nicotinic acid, 70 mg, folic acid, 3 mg, calcium pantothenate, 0.375 mg, antioxidant, 0.15 mg, choline chloride, 105 mg; Co as CoSO_4_, 1 mg; Cu as CuSO_4_·5H_2_O, 155 mg; Fe as FeSO_4_·H_2_O, 145 mg; Mn as MnO, 75 mg; Zn as ZnSO_4_, 125 mg; I as KI, 0.3 mg; Se as Na_2_SeO_3_, 0.3 mg. ^c^ Data are calculated according to NRC (2012) standards [16]. ^d^ SID, standardized ileal digestible values.

**Table 2:** Effects of DF structure and AV on growth performance in pigs.

Items	AV ^1^		β/AX ^2^			Treatments						SEM	*p*-Value				
	L_V_	H_V_	L_β/AX_	M_β/AX_	H_β/AX_	L_V_L_β/AX_	L_V_M_β/AX_	L_V_H_β/AX_	H_V_L_β/AX_	H_V_M_β/AX_	H_V_H_β/AX_		AV	β/AX	AV × β/AX	L	Q
IW (Kg)	47.27	47.16	47.39	46.86	47.39	47.55	46.58	47.67	47.23	47.14	47.11	0.437	0.906	0.860	0.869	0.917	0.997
FW (Kg)	72.32 ^m^	70.27 ^n^	72.41 ^x^	71.29 ^y^	70.19 ^z^	73.58 ^a^	72.32 ^ab^	71.05 ^b^	71.24 ^b^	70.26 ^bc^	69.33 ^c^	0.451	<0.001	0.014	0.166	<0.001	0.119
ADFI (Kg/d)	1.93	1.86	1.92	1.88	1.88	1.97	1.89	1.93	1.88	1.87	1.83	0.016	0.061	0.271	0.427	0.065	0.943
ADG (Kg/d)	0.84 ^m^	0.77 ^n^	0.84 ^x^	0.82 ^xy^	0.76 ^y^	0.87 ^a^	0.86 ^ab^	0.78 ^c^	0.80 ^b^	0.77 ^c^	0.74 ^d^	0.001	<0.001	<0.001	0.021	<0.001	0.059
F/G	2.31 ^n^	2.42 ^m^	2.30 ^y^	2.32 ^y^	2.47 ^x^	2.26 ^c^	2.20 ^d^	2.47 ^a^	2.35 ^b^	2.42 ^ab^	2.47 ^a^	0.014	<0.001	<0.001	0.005	<0.001	0.201

AV: apparent viscosity; β/AX: β-glucan-to-arabinoxylan ratio; IW: initial weight; FW: final weight; ADFI: average daily feed intake; ADG: average daily gain; F/G: feed-to-gain ratio; L_V_L_β/AX_: diet with low apparent viscosity and low β-glucan-to-arabinoxylan ratio; L_V_M_β/AX_: diet with low apparent viscosity and middle β-glucan-to-arabinoxylan ratio; L_V_H_β/AX_: diet with low apparent viscosity and high β-glucan-to-arabinoxylan ratio; H_V_L_β/AX_: diet with high apparent viscosity and low β-glucan-to-arabinoxylan ratio; H_V_M_β/AX_: diet with high apparent viscosity and middle β-glucan-to-arabinoxylan ratio; H_V_H_β/AX_: diet with high apparent viscosity and high β-glucan-to-arabinoxylan ratio; SEM: standard error of the mean; AV × β/AX: linear interaction effects between dietary apparent viscosity and β-glucan-to-arabinoxylan ratio; L: linear; Q: quadratic. ^1^ L_V_ and H_V_ are the two treatment groups under the main effect of AV, representing the low AV group and the high AV group, respectively. ^2^ L_β/AX_, M_β/AX_, and H_β/AX_ are the three treatment groups under the main effect of the β/AX, representing the low β/AX, middle β/AX, and high β/AX group, respectively. ^a–d,m,n,x–z^ Distinct letters denote notable discrepancies among the six dietary treatment groups, among the main effects of AV, and among the main effects of the β/AX, separately (*p* < 0.05).

**Table 3:** Effects of DF structure and AV on carcass traits of pigs.

Items	AV ^1^		β/AX ^2^			Treatments						SEM	*p*-Value				
	L_V_	H_V_	L_β/AX_	M_β/AX_	H_β/AX_	L_V_L_β/AX_	L_V_M_β/AX_	L_V_H_β/AX_	H_V_L_β/AX_	H_V_M_β/AX_	H_V_H_β/AX_		AV	β/AX	AV × β/AX	L	Q
Pre-slaughter weight (kg)	72.32 ^m^	70.27 ^n^	72.41 ^x^	71.29 ^y^	70.19 ^z^	73.58 ^a^	72.32 ^ab^	71.05 ^b^	71.24 ^b^	70.26 ^bc^	69.33 ^c^	0.451	<0.001	0.014	0.166	<0.001	0.119
Carcass weight (kg)	52.03 ^m^	49.67 ^n^	52.21 ^x^	50.86 ^y^	49.48 ^y^	53.38 ^a^	52.09 ^ab^	50.62 ^bc^	51.03 ^b^	49.63 ^c^	48.42 ^d^	0.245	<0.001	0.026	<0.001	0.025	0.324
Dressing percentage (%)	71.94 ^m^	70.69 ^n^	72.09 ^x^	71.34 ^xy^	70.53 ^y^	72.55 ^a^	72.04 ^a^	71.24 ^b^	71.63 ^ab^	70.64 ^c^	69.82 ^d^	0.392	0.045	0.031	<0.001	<0.001	0.752
Loin-eye area (cm^2^)	32.33	31.99	32.55	32.02	31.93	32.73	32.16	32.10	32.38	31.88	31.73	0.271	0.356	0.241	0.536	0.216	0.647
Lean meat percentage (%)	51.76	52.29	51.68	52.01	52.43	51.46	51.58	52.25	51.87	52.41	52.95	0.414	0.054	0.062	0.251	0.057	0.213
Lean meat mass (kg)	37.43	36.84	37.41	37.07	36.92	37.86	37.30	37.12	36.95	36.83	36.72	0.370	0.146	0.259	0.117	0.454	0.532
Fat percentage (%)	8.64 ^m^	7.80 ^n^	8.86 ^x^	8.11 ^y^	7.70 ^z^	9.46 ^a^	8.41 ^b^	8.05 ^bc^	8.26 ^bc^	7.80 ^c^	7.34 ^d^	0.043	0.021	<0.001	<0.001	0.032	0.816
Fat mass (kg)	6.25 ^m^	5.49 ^n^	6.43 ^x^	5.78 ^y^	5.40 ^z^	6.97 ^a^	6.07 ^b^	5.71 ^c^	5.89 ^bc^	5.48 ^cd^	5.09 ^d^	0.021	<0.001	0.015	0.033	<0.001	0.347
Backfat thickness (mm)	12.47 ^m^	11.26 ^n^	12.98 ^x^	11.64 ^y^	10.98 ^z^	13.84 ^a^	12.18 ^b^	11.39 ^c^	12.12 ^b^	11.09 ^c^	10.57 ^d^	0.015	<0.001	0.013	<0.001	<0.001	0.125
Abdominal fat thickness (mm)	14.51 ^m^	12.43 ^n^	14.53 ^x^	13.36 ^y^	12.51 ^z^	15.28 ^a^	14.60 ^ab^	13.65 ^bc^	13.78 ^b^	12.11 ^d^	11.39 ^e^	0.011	0.026	<0.001	<0.001	<0.001	0.437

AV: apparent viscosity; β/AX: β-glucan-to-arabinoxylan ratio; L_V_L_β/AX_: diet with low apparent viscosity and low β-glucan-to-arabinoxylan ratio; L_V_M_β/AX_: diet with low apparent viscosity and middle β-glucan-to-arabinoxylan ratio; L_V_H_β/AX_: diet with low apparent viscosity and high β-glucan-to-arabinoxylan ratio; H_V_L_β/AX_: diet with high apparent viscosity and low β-glucan-to-arabinoxylan ratio; H_V_M_β/AX_: diet with high apparent viscosity and middle β-glucan-to-arabinoxylan ratio; H_V_H_β/AX_: diet with high apparent viscosity and high β-glucan-to-arabinoxylan ratio; SEM: standard error of the mean; AV × β/AX: linear interaction effects between dietary apparent viscosity and β-glucan-to-arabinoxylan ratio; L: linear; Q: quadratic. ^1^ L_V_ and H_V_ are the two treatment groups under the main effect of AV, representing the low AV group and the high AV group, respectively. ^2^ L_β/AX_, M_β/AX_, and H_β/AX_ are the three treatment groups under the main effect of the β/AX, representing the low β/AX, middle β/AX, and high β/AX group, respectively. ^a–e,m,n,x–z^ Distinct letters denote notable discrepancies among the six dietary treatment groups, among the main effects of AV, and among the main effects of the β/AX, separately (*p* < 0.05).

**Table 4:** Effects of DF structure and AV on meat quality.

Items	AV ^1^		β/AX ^2^			Treatments						SEM	*p*-Value				
	L_V_	H_V_	L_β/AX_	M_β/AX_	H_β/AX_	L_V_L_β/AX_	L_V_M_β/AX_	L_V_H_β/AX_	H_V_L_β/AX_	H_V_M_β/AX_	H_V_H_β/AX_		AV	β/AX	AV × β/AX	L	Q
Physical characteristics																	
L*_24 h_	42.21	42.07	41.89	42.63	41.92	41.76	42.63	42.25	42.01	42.62	41.58	0.310	0.463	0.501	0.418	0.325	0.751
a*_24 h_	6.96 ^n^	7.55 ^m^	7.26	7.13	7.38	7.12	6.84	6.92	7.39	7.42	7.83	0.126	0.034	0.630	0.162	0.467	0.856
b*_24 h_	8.48	8.99	8.59	8.62	8.99	8.05	8.46	8.92	9.13	8.78	9.05	0.115	0.061	0.223	0.574	0.263	0.549
pH_45 min_	6.26	6.27	6.24	6.28	6.28	6.24	6.28	6.25	6.23	6.27	6.30	0.073	0.182	0.241	0.643	0.635	0.714
pH_24 h_	5.61	5.59	5.61	5.62	5.60	5.63	5.61	5.59	5.58	5.63	5.60	0.058	0.085	0.160	0.326	0.752	0.883
SF (N)	38.35 ^m^	36.44 ^n^	38.01 ^x^	37.29 ^xy^	36.90 ^y^	38.63 ^a^	38.46 ^a^	37.97 ^b^	37.38 ^c^	36.11 ^cd^	35.83 ^d^	0.483	0.016	0.035	0.021	<0.001	0.273
DL (%)	3.01	2.84	3.07	2.85	2.84	3.16	2.94	2.90	2.98	2.75	2.78	0.059	0.273	0.491	0.648	0.062	0.548
CL (%)	27.88	28.41	27.75	28.14	28.54	27.68	27.64	28.31	27.82	28.64	28.76	0.468	0.358	0.172	0.326	0.784	0.851
MS	2.22	2.25	2.17	2.26	2.29	2.18	2.22	2.27	2.15	2.29	2.31	0.135	0.409	0.456	0.561	0.563	0.784
Chemical characteristics																	
DM (%)	27.99 ^n^	29.33 ^m^	28.09 ^y^	28.67 ^xy^	29.23 ^x^	27.69 ^c^	27.78 ^cb^	28.51 ^b^	28.48 ^b^	29.56 ^ab^	29.94 ^a^	0.261	0.043	0.018	<0.001	0.031	0.615
CP (%)	22.01	24.39	22.71	23.08	23.82	21.54	21.83	22.65	23.87	24.32	24.98	0.382	0.051	0.065	0.073	0.025	0.457
IMF (%)	2.93	3.33	3.00	3.16	3.24	2.85	2.94	2.99	3.15	3.37	3.48	0.267	0.056	0.058	0.064	0.016	0.582

AV: apparent viscosity; β/AX: β-glucan-to-arabinoxylan ratio; L_V_L_β/AX_: diet with low apparent viscosity and low β-glucan-to-arabinoxylan ratio; L_V_M_β/AX_: diet with low apparent viscosity and middle β-glucan-to-arabinoxylan ratio; L_V_H_β/AX_: diet with low apparent viscosity and high β-glucan-to-arabinoxylan ratio; H_V_L_β/AX_: diet with high apparent viscosity and low β-glucan-to-arabinoxylan ratio; H_V_M_β/AX_: diet with high apparent viscosity and middle β-glucan-to-arabinoxylan ratio; H_V_H_β/AX_: diet with high apparent viscosity and high β-glucan-to-arabinoxylan ratio; SEM: standard error of the mean; AV × β/AX: linear interaction effects between dietary apparent viscosity and β-glucan-to-arabinoxylan ratio; L: linear; Q: quadratic; L*_24 h_: lightness at 24 h post-mortem; a*_24 h_: redness at 24 h post-mortem; b*_24 h_: yellowness at 24 h post-mortem; SF: shear force; DL: drip loss; CL: cooking loss; MS: marbling score; DM: dry matter; CP: crude protein; IMF: intramuscular fat. ^1^ L_V_ and H_V_ are the two treatment groups under the main effect of apparent viscosity, representing the low apparent viscosity group and the high apparent viscosity group, respectively. ^2^ L_β/AX_, M_β/AX_, and H_β/AX_ are the three treatment groups under the main effect of the β/AX, representing the low β/AX, middle β/AX, and high β/AX group, respectively. ^a–d,m,n,x,y^ Distinct letters denote notable discrepancies among the six dietary treatment groups, among the main effects of AV, and among the main effects of the β/AX, separately (*p* < 0.05).

**Table 5:** Effects of DF structure and AV on blood biochemistry of pigs.

Items	AV ^1^		β/AX ^2^			Treatments						SEM	*p*-Value				
	L_V_	H_V_	L_β/AX_	M_β/AX_	H_β/AX_	L_V_L_β/AX_	L_V_M_β/AX_	L_V_H_β/AX_	H_V_L_β/AX_	H_V_M_β/AX_	H_V_H_β/AX_		AV	β/AX	AV × β/AX	L	Q
TP (g/L)	62.13	62.33	62.83	60.88	62.98	60.43	61.87	64.10	65.23	59.90	61.87	0.751	0.891	0.434	0.121	0.944	0.237
ALB (g/L)	26.26	27.21	26.82	26.42	26.97	25.37	26.20	27.20	28.27	26.63	26.73	0.362	0.192	0.801	0.167	0.872	0.563
UN (mmol/L)	2.17	2.36	2.72	2.08	2.00	2.87	1.84	1.80	2.57	2.32	2.19	0.155	0.525	0.125	0.506	0.081	0.364
GLU (mmol/L)	5.38 ^m^	5.01 ^n^	5.55 ^x^	5.16 ^y^	5.06 ^z^	5.54 ^a^	5.32 ^ab^	5.25 ^b^	5.16 ^b^	4.99 ^c^	4.87 ^d^	0.061	0.032	0.042	0.031	0.032	0.438
HDL-C (mmol/L)	0.95	1.09	0.92 ^y^	1.03 ^y^	1.11 ^x^	0.86 ^c^	0.96 ^b^	1.04 ^b^	0.98 ^b^	1.09 ^ab^	1.18 ^a^	0.023	0.076	0.014	0.047	0.040	0.427
LDL-C (mmol/L)	1.01	0.98	1.12	0.97	0.91	1.12	0.98	0.94	1.11	0.96	0.88	0.046	0.262	0.057	0.624	0.087	0.142
TG (mmol/L)	0.41 ^m^	0.29 ^n^	0.41	0.33	0.31	0.44 ^a^	0.40 ^ab^	0.39 ^ab^	0.37 ^bc^	0.26 ^c^	0.24 ^c^	0.021	0.026	0.054	0.072	0.053	0.436
TC (mmol/L)	2.40 ^m^	2.18 ^n^	2.49 ^x^	2.28 ^xy^	2.12 ^y^	2.60 ^a^	2.45 ^ab^	2.21 ^bc^	2.37 ^b^	2.14 ^cd^	2.03 ^d^	0.053	0.045	0.013	0.011	0.027	0.162
LDH (U/L)	574.41	552.99	585.17	516.43	589.5	516.60	552.03	654.60	653.73	480.83	524.40	21.924	0.553	0.206	0.182	0.941	0.141

AV: apparent viscosity; β/AX: β-glucan-to-arabinoxylan ratio; L_V_L_β/AX_: diet with low apparent viscosity and low β-glucan-to-arabinoxylan ratio; L_V_M_β/AX_: diet with low apparent viscosity and middle β-glucan-to-arabinoxylan ratio; L_V_H_β/AX_: diet with low apparent viscosity and high β-glucan-to-arabinoxylan ratio; H_V_L_β/AX_: diet with high apparent viscosity and low β-glucan-to-arabinoxylan ratio; H_V_M_β/AX_: diet with high apparent viscosity and middle β-glucan-to-arabinoxylan ratio; H_V_H_β/AX_: diet with high apparent viscosity and high β-glucan-to-arabinoxylan ratio; SEM: standard error of the mean; AV × β/AX: linear interaction effects between dietary apparent viscosity and β-glucan-to-arabinoxylan ratio; L: linear; Q: quadratic; TP: total protein; ALB: albumin; UN: urea nitrogen; GLU: glucose; HDL-C: high-density lipoprotein cholesterol; LDL-C: low-density lipoprotein cholesterol; TG: triglycerides; TC: total cholesterol; LDH: lactate dehydrogenase. ^1^ L_V_ and H_V_ are the two treatment groups under the main effect of apparent viscosity, representing the low apparent viscosity group and the high apparent viscosity group, respectively. ^2^ L_β/AX_, M_β/AX_, and H_β/AX_ are the three treatment groups under the main effect of the β/AX, representing the low β/AX, middle β/AX, and high β/AX group, respectively. ^a–d,m,n,x–z^ Distinct letters denote notable discrepancies among the six dietary treatment groups, among the main effects of AV, and among the main effects of the β/AX, separately (*p* < 0.05).

**Table 6:** Effects of DF structure and AV on the production of short-chain fatty acids in the middle colon of pigs.

Items	AV ^1^		β/AX ^2^			Treatments						SEM	*p*-Value				
	L_V_	H_V_	L_β/AX_	M_β/AX_	H_β/AX_	L_V_L_β/AX_	L_V_M_β/AX_	L_V_H_β/AX_	H_V_L_β/AX_	H_V_M_β/AX_	H_V_H_β/AX_		AV	β/AX	AV × β/AX	L	Q
SCFA in the middle colon, μmol/g																	
Acetate	31.98 ^n^	34.48 ^m^	32.56 ^y^	32.93 ^y^	34.18 ^x^	31.27 ^b^	31.82 ^b^	32.81 ^ab^	33.85 ^ab^	34.04 ^ab^	35.54 ^a^	0.415	0.031	0.043	0.257	0.118	0.618
Propionate	12.65	13.72	12.97	13.40	13.19	12.29	12.98	12.68	13.64	13.82	13.70	0.274	0.063	0.816	0.532	0.821	0.594
Butyrate	5.91 ^n^	6.93 ^m^	5.72 ^z^	6.46 ^y^	7.08 ^x^	5.35 ^e^	6.05 ^d^	6.33 ^c^	6.09 ^d^	6.87 ^b^	7.84 ^a^	0.133	<0.001	0.013	<0.001	<0.001	0.773
Valerate	0.83	0.88	0.79 ^y^	0.88 ^x^	0.90 ^x^	0.74 ^b^	0.86 ^ab^	0.89 ^a^	0.83 ^ab^	0.90^a^	0.91 ^a^	0.068	0.133	0.019	0.622	0.069	0.280
Isobutyrate	0.45	0.41	0.46	0.43	0.40	0.48	0.47	0.41	0.44	0.40	0.40	0.029	0.077	0.246	0.371	0.464	0.800
Isovalerate	0.43	0.40	0.44	0.39	0.39	0.47	0.41	0.40	0.41	0.38	0.37	0.026	0.101	0.152	0.519	0.352	0.181
TSCFA	52.24 ^n^	56.81 ^m^	52.93 ^z^	54.50 ^y^	56.14 ^x^	50.60 ^d^	52.58 ^cd^	53.53 ^bcd^	55.26 ^abc^	56.42 ^ab^	58.76 ^a^	0.635	<0.001	0.038	0.043	0.040	0.970

AV: apparent viscosity; β/AX: β-glucan-to-arabinoxylan ratio; L_V_L_β/AX_: diet with low apparent viscosity and low β-glucan-to-arabinoxylan ratio; L_V_M_β/AX_: diet with low apparent viscosity and middle β-glucan-to-arabinoxylan ratio; L_V_H_β/AX_: diet with low apparent viscosity and high β-glucan-to-arabinoxylan ratio; H_V_L_β/AX_: diet with high apparent viscosity and low β-glucan-to-arabinoxylan ratio; H_V_M_β/AX_: diet with high apparent viscosity and middle β-glucan-to-arabinoxylan ratio; H_V_H_β/AX_: diet with high apparent viscosity and high β-glucan-to-arabinoxylan ratio; SEM: standard error of the mean; AV × β/AX: linear interaction effects between dietary apparent viscosity and β-glucan-to-arabinoxylan ratio; L: linear; Q: quadratic; SCFA: short-chain fatty acids; TSCFA: total short-chain fatty acids. ^1^ L_V_ and H_V_ are the two treatment groups under the main effect of apparent viscosity, representing the low apparent viscosity group and the high apparent viscosity group, respectively. ^2^ L_β/AX_, M_β/AX_, and H_β/AX_ are the three treatment groups under the main effect of the β/AX, representing the low β/AX, middle β/AX, and high β/AX group, respectively. ^a–e,m,n,x–z^ Distinct letters denote notable discrepancies among the six dietary treatment groups, among the main effects of AV, and among the main effects of the β/AX, separately (*p* < 0.05).

## Data Availability

Data will be made available upon request.

## Supplementary Materials

The following supporting information can be downloaded at: https://www.mdpi.com/article/10.3390/ani15223310/s1. Figure S1: Effects of dietary fiber structure and apparent viscosity on plasma GLP-1 and PYY in pigs; Figure S2: Effects of the chemical structure and physical properties of dietary fiber on the α-diversity of gut microbiota; Figure S3. Differential analysis of the top 6 genera in terms of relative abundance in the middle colon; Table S1: Nutrients and physical properties of the feeds (as-fed basis); Table S2: Primer sequences used for real-time quantitative PCR analysis.

## Abbreviations

The following abbreviations are used in this manuscript:

DF	Dietary fiber
SDF	Soluble dietary fiber
IDF	Insoluble dietary fiber
SCFA	Short-chain fatty acids
AV	Apparent viscosity
β/AX	β-glucan-to-arabinoxylan ratio
IW	Initial weight
FW	Final weight
ADG	Average daily gain
ADFI	Average daily feed intake
F/G	Feed-to-gain ratio
LDM	*Longissimus dorsi* muscle
DM	Dry matter
CP	Crude protein
IMF	Intramuscular fat
LDH	Lactate dehydrogenase
TG	Triglycerides
TC	Total cholesterol
LDL-C	Low-density lipoprotein cholesterol
HDL-C	High-density lipoprotein cholesterol
GLU	Glucose
UN	Urea nitrogen
ALB	Albumin
TP	Total protein
GLP-1	Glucagon-like peptide-1
PYY	Peptide YY
QC	Quality control
ASVs	Amplicon sequence variants
SREBP-1C	Sterol regulatory element-binding protein-1C
ACC	Acetyl-CoA carboxylase
FAS	Fatty acid synthase
AMPK	AMP-activated protein kinase
CPT1	Carnitine palmitoyltransferase 1
PPAR-α	Peroxisome proliferator-activated receptor alpha
TSCFA	Total short-chain fatty acids
