# Supplementary material for: Effect of the Interaction Between Dietary Fiber Structure and Apparent Viscosity on the Production Performance of Growing Pigs
Source: Animals (Basel). 2025 Nov 17;15(22):3310. doi: 10.3390/ani15223310 (PMC12649407; doi:10.3390/ani15223310)
Supplement: Supplementary file 1 [file animals-15-03310-s001.zip › animals-3976227-supplementary.pdf]

Article

Effect of the Interaction Between Dietary Fiber Structure and Apparent Viscosity on the Production Performance of Growing Pigs

Feng Yong [1,2,3,4 †], Huijuan Li [1,2,3,4 †], Bing Hu [1,2,3,4], Bo Liu [1,2,3,4], Rui Han [1,2,3,4,*] and Dongsheng Che [1,2,3,4,*]

[1] Key Laboratory of Animal Production, Product Quality and Security, Ministry of Education
[2] Jilin Provincial Key Laboratory of Animal Nutrition and Feed Science
[3] Jilin Provincial Science and Technology Innovation Center of Pig Industry Technology
[4] College of Animal Science and Technology, Jilin Agricultural University, Changchun 130118, PR China
* Correspondence: chedongsheng@jlau.edu.cn (D. C.); hanrui0409@163.com (R. H.)
† These authors contributed equally to this work

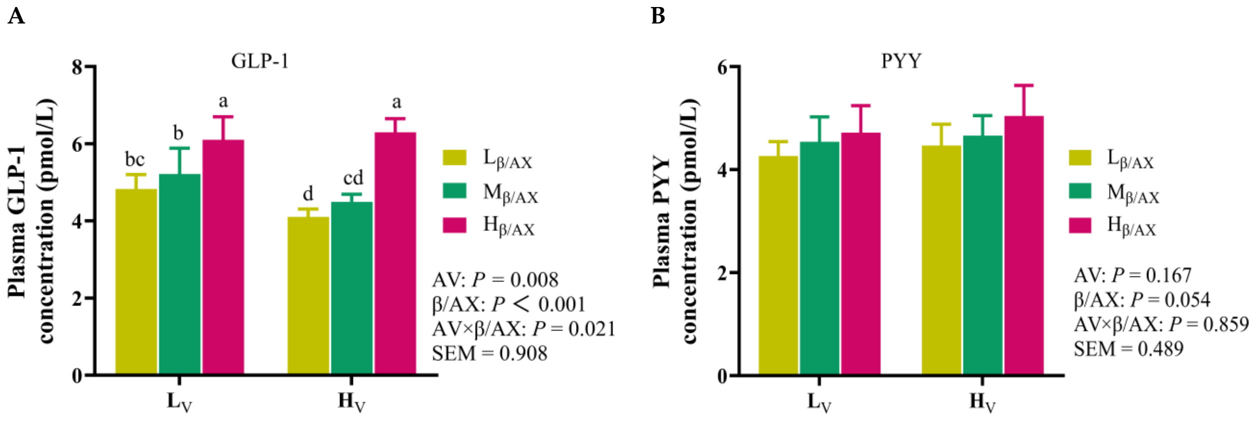

Figure S1. Effects of dietary fiber structure and apparent viscosity on plasma GLP-1 and PYY in pigs**.** The bar chart presents the differential analysis of the concentration of plasma GLP-1 (A) and PYY (B). AV: apparent viscosity; β/AX: β-glucan-to-arabinoxylan ratio; AV × β/AX: interaction effect between the dietary apparent viscosity and β-glucan-to-arabinoxylan ratio; L_V: low apparent viscosity; H_V: high apparent viscosity; $L_{β/AX}$: low β-glucan-to-arabinoxylan ratio; $M_{β/AX}$: medium β-glucan-to-arabinoxylan ratio; $H_{β/AX}$: high β-glucan-to-arabinoxylan ratio; SEM: standard error of the mean; GLP-1: glucagon-like peptide-1; PYY: peptide YY. Data were presented as Mean ± SD. Different lowercase letters indicate significant differences between groups ($P < 0.05$).

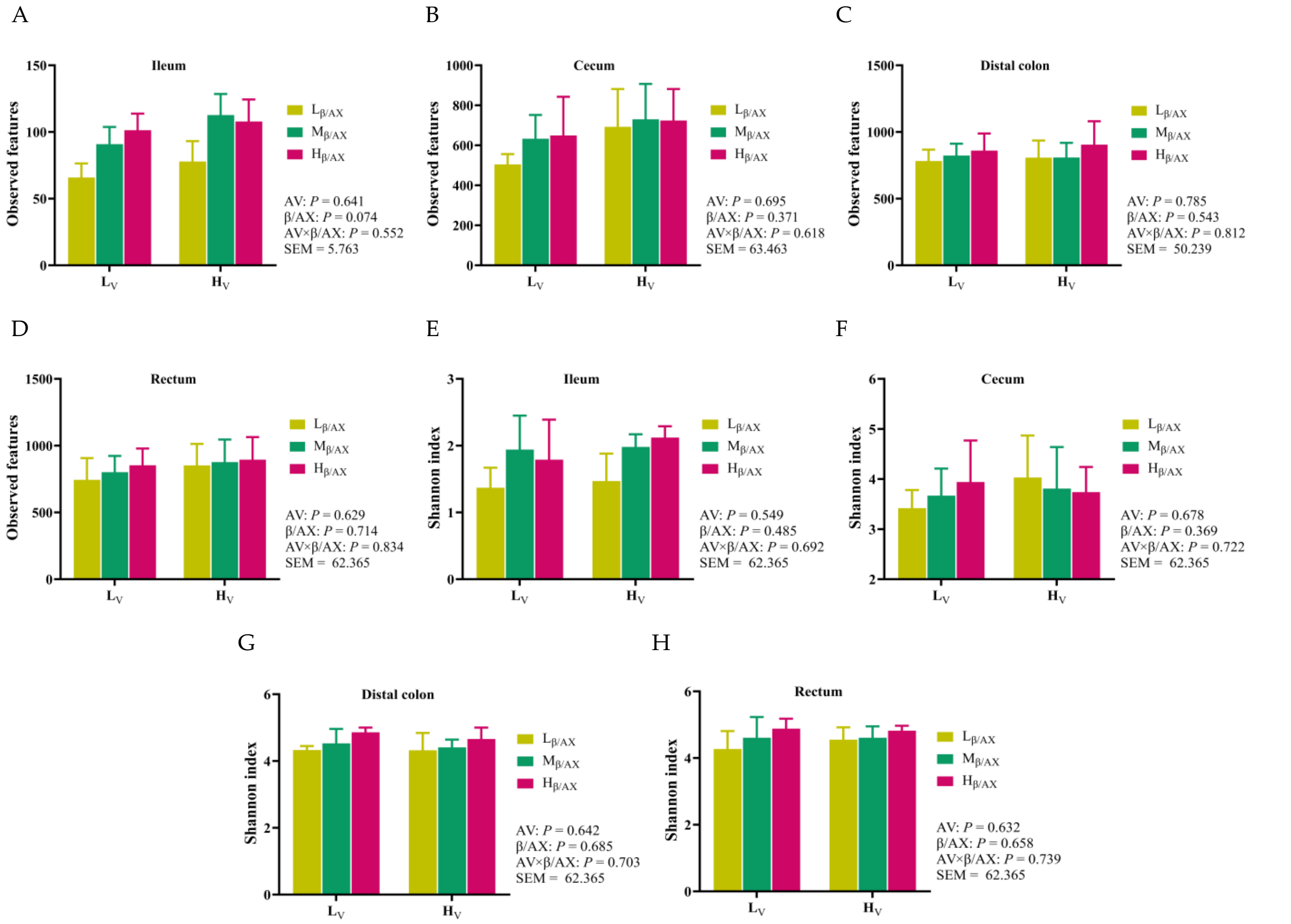

Figure S2. Effects of the chemical structure and physical properties of dietary fiber on the α-diversity of gut microbiota. Panels A-D show the number of observed microbial species in the ileum, cecum, distal colon, and rectum, respectively, while Panels E-H illustrate the Shannon index of microbes in the aforementioned intestinal segments (ileum, cecum, distal colon, and rectum) in sequence. AV: apparent viscosity; β/AX: β-glucan-to-arabinoxylan ratio; AV × β/AX: interaction effect between the dietary apparent viscosity and β-glucan-to-arabinoxylan ratio; L_V: low apparent viscosity; H_V: high apparent viscosity; $L_{\beta/AX}$: low β-glucan-to-arabinoxylan ratio; $M_{\beta/AX}$: medium β-glucan-to-arabinoxylan ratio; $H_{\beta/AX}$: high β-glucan-to-arabinoxylan ratio; SEM: standard error of the mean.

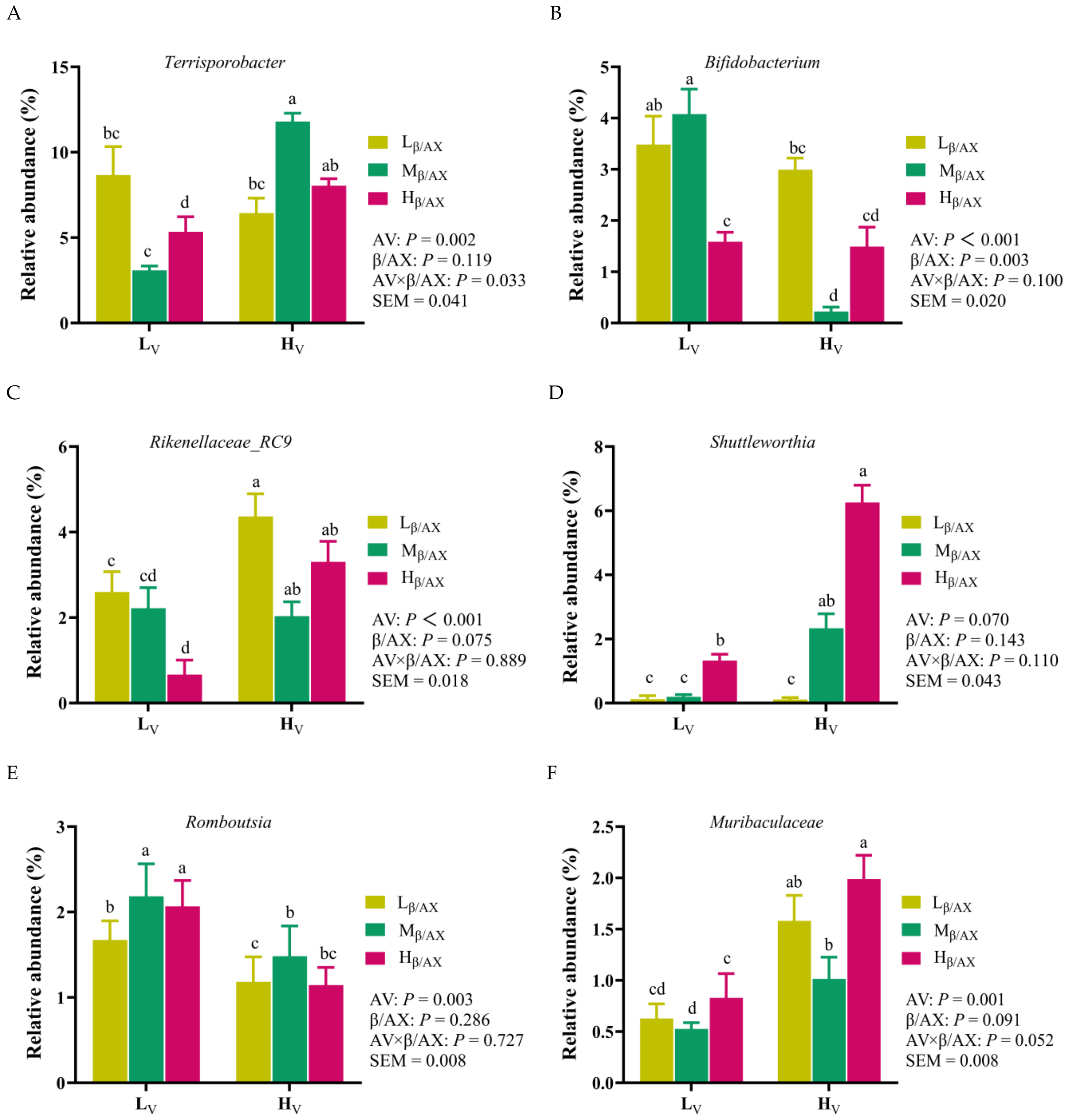

Figure S3. Differential analysis of the top 6 genera in terms of relative abundance in the middle colon. The bar chart shows the differences in *Terrisporobacter* (A), *Bifidobacterium* (B), *Rikenellaceae_RC9* (C), *Shuttleworthia* (D), *Roumboutsia* (E), and *Muribaculaceae* (F). AV: apparent viscosity; β/AX: β-glucan-to-arabinoxylan ratio; AV × β/AX: interaction effect between the dietary apparent viscosity and β-glucan-to-arabinoxylan ratio; L_V: low apparent viscosity; H_V: high apparent viscosity; $L_{\beta/AX}$: low β-glucan-to-arabinoxylan ratio; $M_{\beta/AX}$: medium β-glucan-to-arabinoxylan ratio; $H_{\beta/AX}$: high β-glucan-to-arabinoxylan ratio; SEM, standard error of the mean. Data were presented as Mean ± SD. Different lowercase letters indicate significant differences between groups ($P < 0.05$).

Table S1. Nutrients and physical properties of the feeds (as-fed basis).

Items	Corn	Wheat	Barley	Corn husk	Soybean husk	Soybean meal	Rapeseed meal	Sugar beet pulp	Corn gluten meal	Corn DDGS
DM (%)	89.80	88.85	90.74	94.38	93.58	91.94	92.95	93.29	94.20	90.73
OM (%)	88.78	87.39	88.45	91.17	89.33	85.50	85.34	87.91	92.76	86.45
EE (%)	3.75	1.18	2.83	3.30	1.47	1.57	2.27	0.54	0.93	4.51
CP (%)	10.09	12.78	11.53	8.11	9.84	49.79	38.20	9.88	57.66	28.26
Ash (%)	1.02	1.46	2.29	3.21	4.25	6.44	7.61	5.38	1.44	4.28
CF (%)	2.41	2.65	5.45	21.60	40.25	6.14	10.77	28.93	3.07	11.65
NFE (%)	72.19	70.66	67.62	55.47	37.78	30.34	40.03	47.29	26.11	47.11
TDF (%)	12.09	11.91	20.45	57.83	54.39	18.59	32.23	69.51	8.23	32.66
SDF (%)	1.39	1.66	5.57	6.17	9.61	2.44	7.83	29.30	1.60	3.76
IDF (%)	10.24	9.73	14.66	51.66	44.78	16.15	24.4	40.21	6.63	29.90
ADF (%)	2.62	2.25	5.66	14.14	45.95	5.37	22.15	22.33	1.35	10.25
NDF (%)	11.33	16.27	23.72	49.47	64.00	12.09	32.61	37.53	4.54	30.07
Cellulose (%)	1.88	0.86	4.36	7.45	40.36	4.52	14.14	20.25	0.74	5.21
Lignin (%)	0.74	1.39	1.30	6.69	5.59	0.85	7.75	2.08	0.61	5.04
NSP (%)	11.35	10.52	19.15	51.14	48.80	17.74	24.48	67.43	7.62	27.62
β-Glucan (%)	0.11	0.81	6.66	2.32	0.42	0.61	0.83	0.97	0.33	6.26
Arabinose (%)	2.27	0.94	2.32	0.61	3.21	0.31	0.29	18.11	0.58	4.99
Xylose (%)	3.27	3.24	3.17	3.25	3.14	3.51	3.25	2.98	3.20	3.28
Arabinoxylan (%)	6.20	8.62	7.90	19.78	9.26	2.32	1.72	19.87	1.25	13.12
Mannose (%)	0.20	0.00	0.20	0.40	3.00	1.30	0.60	1.52	0.30	2.00
Galactose (%)	0.63	0.31	0.19	1.83	2.95	3.82	1.66	6.45	0.66	5.45
Glucose (%)	2.57	2.48	1.20	1.27	0.31	0.74	2.07	1.23	1.90	1.69

Cont. Table S1

Items	Corn	Wheat	Barley	Corn husk	Soybean husk	Soybean meal	Rapeseed meal	Sugar beet pulp	Corn gluten meal	Corn DDGS
Rhamnose (%)	-	-	-	-	0.83	0.70	1.96	1.35	-	-
Uronic acid (%)	0.71	0.21	0.20	3.84	1.97	5.00	6.64	33.26	0.51	5.10
Fructan (%)	0.18	1.48	0.40	1.91	1.04	-	-	0.94	0.20	0.12
β-Glucan-to-arabinoxylan ratios	0.02	0.09	0.84	0.12	0.05	0.26	0.48	0.05	0.27	0.48
Ca (%)	0.02	0.06	0.03	0.15	0.97	0.39	0.75	0.81	0.02	0.10
TP (%)	0.58	0.21	0.34	0.50	0.28	0.66	0.87	0.15	0.09	0.73
GE (MJ/kg)	16.51	15.88	16.55	18.17	15.82	17.67	17.70	15.96	20.81	18.17
Apparent viscosity (cP)	1.07	1.12	1.31	1.14	1.25	1.00	1.32	2.12	1.10	1.02

DM: dry matter; OM: organic matter; EE: ether extract; CP: crude protein; CF: crude fiber; NFE: nitrogen-free extract; TDF: total dietary fiber; SDF: soluble dietary fiber; IDF: insoluble dietary fiber; ADF: acid detergent fiber; NDF: neutral detergent fiber; NSP: non-starch polysaccharides; TP: total phosphorus; GE: gross energy; Corn DDGS: corn distillers dried grains with solubles.

Table S2. Primer sequences used for real-time quantitative PCR analysis.

Genes	Accession no.	Primer sequences (5' - 3')	Size (bp)
AMPK	NM_001167633.1	F: GGTGAAAATCGGCCACTACA<br>R: TTGCCAACCTTCACTTTGCC	72
PPARα	NM_001044526.1	F: GGCTGCTATCATTTGGTGCG<br>R: GCACGATACCCTCCTGCATT	80
CPT1	XM_021091195.1	F: CCACTATGACCCGGAAGACG<br>R: TTGAACGCGATGAGGGTGAA	111
SREBP-1c	NM_214157.1	F: CTGCTGACCGACATCGAAGA<br>R: GGAGCTCATGGTGGAAGGAG	168
FAS	NM_213839.1	F: TGGGTTCTCCTGTCACTGGT<br>R: CAGCATGTTTCCGTTTGCCA	196
ACC	XM_021066233.1	F: GGATGAACCGTCTCCCTTGG<br>R: CCAGCTTCACCAGGTTGCTA	116
β-Actin	XM_021086047.1	F: GGACTTCGAGCAGGAGATGG<br>R: AGGAAGGAGGGCTGGAAGAG	138

AMPK: AMP-activated protein kinase; PPAR-α: proliferator-activated receptor alpha; CPT1: carnitine palmitoyltransferase 1; SREBP-1C: sterol regulatory element-binding protein-1C; FAS: fatty acid synthase; ACC: acetyl-CoA carboxylase ; β-actin: Beta-actin; bp: base pair.
